# The Addition of Vascular Calcification Scores to Traditional Risk Factors Improves Cardiovascular Risk Assessment in Patients with Chronic Kidney Disease

**DOI:** 10.1371/journal.pone.0131707

**Published:** 2015-07-16

**Authors:** Sophie Liabeuf, Lucie Desjardins, Momar Diouf, Mohamed Temmar, Cédric Renard, Gabriel Choukroun, Ziad A. Massy

**Affiliations:** 1 INSERM U1088, Jules Verne University of Picardy Amiens, France; 2 Clinical Research Centre and Division of Clinical Pharmacology, Amiens University Hospital and the Jules Verne University of Picardy, Amiens, France; 3 Department of Biostatistics, Amiens University Hospital, Amiens, France; 4 Division of Radiology, Amiens University Hospital, Amiens, France; 5 Division of Nephrology, Amiens University Hospital, Amiens, France; 6 Division of Nephrology, Ambroise Paré University Hospital, APHP, University of Paris Ouest-Versailles-Saint-Quentin-en-Yvelines (UVSQ), Boulogne-Billancourt/Paris, France; Brigham and Women's Hospital, Harvard Medical School, UNITED STATES

## Abstract

**Background:**

Although a variety of non-invasive methods for measuring cardiovascular (CV) risk (such as carotid intima media thickness, pulse wave velocity (PWV), coronary artery and aortic calcification scores (measured either by CT scan or X-ray) and the ankle brachial index (ABI)) have been evaluated separately in chronic kidney disease (CKD) cohorts, few studies have evaluated these methods simultaneously. Here, we looked at whether the addition of non-invasive methods to traditional risk factors (TRFs) improves prediction of the CV risk in patients at different CKD stages.

**Methods:**

We performed a prospective, observational study of the relationship between the outputs of non-invasive measurement methods on one hand and mortality and CV outcomes in 143 patients at different CKD stages on the other. During the follow-up period, 44 patients died and 30 CV events were recorded. We used Cox models to calculate the relative risk for outcomes. To assess the putative clinical value of each method, we also determined the categorical net reclassification improvement (NRI) and the integrated discrimination improvement.

**Results:**

Vascular calcification, PWV and ABI predicted all-cause mortality and CV events in univariate analyses. However, after adjustment for TRFs, only aortic and coronary artery calcification scores were found to be significant, independent variables. Moreover, the addition of coronary artery calcification scores to TRFs improved the specificity of prediction by 20%.

**Conclusion:**

The addition of vascular calcification scores (especially the coronary artery calcification score) to TRFs appears to improve CV risk assessment in a CKD population.

## Introduction

Cardiovascular disease (CVD) has become a major cause of morbidity and a leading contributor to mortality [1]. In the general population, most CVD is caused by traditional risk factors (TRFs) that can be controlled, treated or modified (such as hypertension, tobacco use, diabetes, lipid levels). Of course, some risk factors cannot be changed (such as age, gender, and family history) [2].

Chronic kidney disease (CKD) is associated with a high incidence of cardiovascular (CV) mortality [3]. Indeed, a decrease in the glomerular filtration rate is associated with an increase in the frequency of CV events and mortality [4, 5]. The mechanisms involved in CVD in CKD patients are complex. Indeed, TRFs fail to fully explain the elevated CV risk in these patients [6]. Tools capable of improving CV risk assessment (relative to TRFs alone) are clearly necessary in CKD patients. Previous studies have evaluated the prognostic value of the intima media thickness (IMT) in CKD patients and hemodialysis patients [7–11]. Aortic stiffness (as evaluated from the carotid femoral pulse wave velocity, PWV) has also emerged as a strong predictor of all-cause and CV mortality in CKD patients [12, 13]. Chronic kidney disease patients with either an abnormally high ankle brachial index (ABI) or an ABI below 0.9 had a poor prognosis for all-cause and CV mortality [14, 15]. The prevalence of vascular calcification increases steadily with the CKD stage and peaks in stage 5D patients [16]. The presence of vascular calcification has been associated with a several-fold increase in the risk of morbidity and mortality in CKD stage 5D patients [17, 18]. Moreover, the Kidney Disease Improving Global Outcomes international clinical practice guideline suggests that CKD stages 3–5D patients with known vascular/valvular calcification need to “be considered as having the highest possible CV risk” [19]. The impacts of several different non-invasive measurement methods have been separately evaluated in CKD cohorts. However, few studies have evaluated the various methods simultaneously [20, 21]. Recently, Matsushita et al.'s analysis of a large group of CKD and non-CKD subjects from the Multi-Ethnic Study of Atherosclerosis cohort demonstrated that non-invasive measurement methods improved overall cardiovascular prediction for the coronary artery calcium score [20]. However, the researchers did not evaluate arterial stiffness or calculate an aortic calcification score. The objective of the present study was therefore to determine whether the addition of non-invasive indices (such as IMT, PWV, ABI, and vascular calcification) to TRFs can improve risk prediction in well characterized, closely monitored patients at different CKD stages. The secondary objective was to determine which of the non-invasive methods had the greatest predictive power regarding the outcomes studied here.

## Methods

### Patient selection

Over an 18-month period (from January 2006 to June 2007), 143 patients at various stages of CKD were recruited by the Nephrology Department at Amiens Hospital (Amiens, France). The present study was approved by the local investigational review board (*Comité de Protection des Personnes Nord-Ouest II*, approval number 06H3) and written, informed consent was provided from all participants, in line with the tenets of the Declaration of Helsinki.

The inclusion criteria included age over 40, close monitoring by a nephrologist, and CKD, as defined by (i) two estimated glomerular filtration rates < 90 ml/min per 1.73 m^2^ (calculated according to Cockcroft and Gault's equation) at a 3– to 6–month interval [22] or (ii) the use of hemodialysis.

The main exclusion criteria were the presence of chronic inflammatory disease, atrial fibrillation, complete heart block, abdominal aorta aneurysm, an aortic and/or femoral artery prosthesis, primary hyperparathyroidism, kidney transplantation or any acute CV event in the three months prior to screening.

### Study protocol

One hundred and forty-three patients were day-hospitalized for research purposes: laboratory blood tests, blood pressure measurements, IMT, PWV, and ABI determinations, a lateral lumbar X-ray and a multislice spiral computed tomography (CT) scan (to determine CV calcification).

Hemodialysis patients were examined on a dialysis-free day or, if this was not possible, the morning before a dialysis session. These patients underwent a four-hour, standard hemodialysis session three times a week. A patient interview focused on comorbidities, the disease history (especially previous CV events), concomitant medications, and TRFs (age, gender, diabetes, triglyceride levels, hypertension, and smoking status). The patient’s medical files were reviewed in order to gather more exhaustive information. Previous CVD was defined as a history of any of the following events in the patient's medical records: myocardial infarction, stroke, heart failure, angina pectoris or surgical procedures for angina or coronary/peripheral artery disease (including percutaneous transluminal angioplasty). Hypertension was defined as treatment with at least one antihypertensive agent. Diabetes mellitus was defined as treatment with insulin or oral antidiabetic medications.

### Laboratory tests

Blood samples were collected on the morning of the study day (before any other investigations), prepared and then stored at -80°C. All assays were performed on subsequently thawed plasma or serum samples.

Serum total calcium, phosphate, albumin, LDL cholesterol, hemoglobin, creatinine and C-reactive protein levels were measured in an on-site biochemistry laboratory with standard auto-analyzer techniques (the Modular IIP system from Roche Diagnostics, Basel, Switzerland). The laboratory's normative range of phosphate concentrations was 0.8–1.45 mmol/l. Serum intact parathyroid hormone and 25 hydroxyvitamin D levels were assayed with a chemiluminometric immunoassay (the Liaison N-tact PTH CLIA and the Liaison 25 OH vitamin D TOTAL CLIA [which measures both D2 and D3], Diasorin, Stillwater, MN). Serum 1.25 (OH)_2_ vitamin D levels were determined in an RIA (^125^I RIA, Diasorin). Serum cystatin C (Cys C) levels were determined by immunonephelometry (N latex Cystatin C, Siemens, Erlangen, Germany) and serum creatinine (Scr) levels were measured using the Jaffe method based on standard isotope-dilution mass spectrometry (*IDMS*). The Chronic Kidney Disease Epidemiology Collaboration equation was then used to calculate a more accurate estimate of the glomerular filtration rate in all non-dialyzed patients [23]: 177.6 x Scr^-0.62^ x Cys C^-0.57^ x age^-0.20^ x (0.82 if female). Next, patients were classified into CKD stages according to the National Kidney Foundation’s Kidney Disease Outcomes Quality Initiative [23].

### Evaluation of the intima media thickness

The IMT was measured semi-automatically from carotid ultrasound data using dedicated software (M'Ath, Intelligence in Medical Technologies SARL, Paris, France). All measurements were performed by the same sonographer. Using an automated edge detection algorithm, the IMT was measured along a 10 mm-long segment of the far wall of the common carotid artery (CCA, at a site free of any discrete plaques) and corresponded to the distance between the lumen-intima interface and the media-adventitia interface. A mean of 50 measurements were automatically performed on each image (two images per body side) and on each body side (left and right). The greatest measured values for the right and left CCA-IMTs were used in the analysis. Data on reproducibility have been previously published by our group; the correlation coefficient for repeated readings of the CCA-IMT was 0.96 [24].

### Evaluation of the PWV

The PWV between the carotid and femoral arteries was determined transcutaneously by trained physicians, using an automatic, dedicated, validated device (Complior Colson, Createch Industrie, Massy, France), as previously described [16, 24]. The velocity was calculated as the distance L between the two recording sites (measured along the body surface), divided by the time interval t between the feet of the flow waves (i.e. PWV = L/t) [25]. To avoid possible methodological bias, a single senior investigator (MT) performed the PWV measurements. All measurements were performed twice (to confirm reproducibility) and the mean value was subsequently analyzed. Data on the validation of this automatic method and its reproducibility have been published previously; the intra-observer repeatability coefficient was 0.93 and the interobserver reproducibility coefficient was 0.89 [25].

### Calculation of the ankle-brachial index

The ABI was calculated after the participant had rested in the supine position for 5 minutes. A hand-held Doppler probe was used to measure the systolic pressures at the right brachial artery, right posterior tibial artery, left posterior tibial artery and left brachial artery (in that order). According to a previously validated method, the ABI was calculated as the ratio of brachial to ankle artery pressure for each leg. The lower of the two values was subsequently analyzed (unless the ABI was 1.3 or more, in which case the higher value was selected).[26]

### Abdominal aorta imaging with plain radiography

A technique similar to that described by Kauppila et al. [27] was used to obtain images of the lower abdominal aorta and thus determine the aortic calcification score. On a plain radiograph of the abdomen (in the sagittal plane), the aorta was identified as a tubular structure passing in front of the anterior surface of the spine. We used a semi-quantitative scoring system; only the abdominal aorta segments in front of the first to fourth lumbar vertebrae were considered. Points were assigned on a 0–3 scale (0: absence; 1: small; 2: moderate; 3: large), according to the length of each calcified plaque identified along the length of the lumbar vertebrae under consideration. Hence, the score could vary from 0 (no calcification) to 24 (maximal calcification). The score was noted independently by two investigators (SL and LD). In the event of disagreement between the scorers, a consensus was reached for all radiographs. To validate the reproducibility of our vascular calcification scoring, 73 randomly selected radiographies were scored blindly by the two independent readers. The calcification scores were compared by calculating Pearson's correlation coefficient (r = 0.925, p = 0.01); this value demonstrates that the calcification scores were correctly assessed on the basis of our chosen criteria.

### Multislice spiral CT

In order to quantify the presence and extent of aortic and coronary artery calcifications, each patient underwent a multislice spiral CT scan. All examinations were performed with a 64-detector CT scanner (Lightspeed VCT, GE Healthcare, Milwaukee, WI). Detailed technical information on the procedure has been provided elsewhere[16]. Briefly, the total aortic calcification volume was obtained by calculating the sum of all voxels in the remaining volume after thresholding. The abdominal aorta calcification score was calculated as follows: [(total calcification volume) / (aorta wall surface area) x 100]. The Agatston score [28] was used to quantify coronary artery calcification.

### Follow-up

Patients were followed up prospectively for the occurrence of non-fatal CV events or all-cause death (whichever occurred first). A total of 64 events (composite endpoints) were analyzed. Cardiovascular-related hospitalizations and deaths were collected prospectively every six months and adjudicated by two nephrologists. In each case, the patient's medical records were reviewed and the cause of death or the CV event was assigned on the basis of all the available clinical information. For out-of-hospital deaths, the patient’s family doctor was interviewed to obtain pertinent information on the cause. The following criteria were used for the adjudication: angina pectoris was defined as symptoms with objective evidence of ischemia (electrocardiogram or coronary artery angiography); adjudication as congestive heart failure had to be supported by a physician's diagnosis or treatment for heart failure, pulmonary congestion or cardiac dysfunction.

Cardiovascular mortality was defined as any death directly to CV system dysfunction (stroke, myocardial infarction, congestive heart failure or sudden death). Non-fatal CV events were defined as systemic CV dysfunction (stroke, angina pectoris, myocardial infarction, congestive cardiac failure, peripheral ischemia or new-onset arrhythmia) or surgical procedures for angina or coronary/peripheral artery disease. When several events occurred in the same patient, only the time to the first event was considered in our analysis.

### Statistical analysis

Data were expressed as the mean ± SD, median and range or frequency, as appropriate. In the study population, patients were stratified according to the presence or absence of events. Intergroup comparisons were performed with a chi-squared test for categorical variables and Student’s t test or the Mann-Whitney test for continuous variables. The Kaplan-Meier method was used to estimate percentage survival as a function of the presence or absence of the composite factor (a CV event or death). Survival curves were compared in a log-rank test. Putative associations between clinical factors, biological factors and mortality were assessed in univariate and multivariate Cox models. The proportional hazard assumption was checked with the Kolmogorov-type supremum test. In the multivariate analysis, the initial model included TRFs only: age, gender, diabetes, triglyceride level, hypertension, and smoking status. In the additional models (Models 2 to 7), the TRFs were supplemented with the various non-invasive measurements. The discriminant ability of the seven Cox models was assessed with Harrell’s C-index [29], which estimates the proportion of correct predictions (i.e. the proportions of patients with better staging and better survival). The C-index varies from 0.5 (no discrimination) to 1 (perfect discrimination). The net reclassification improvement (NRI) was estimated for predefined risk categories of composite criteria (<10%, 10%-19% and ≥20%), based on existing clinical thresholds for cardiovascular disease [30]. Lastly, the integrated discrimination improvement (IDI) was calculated [31]. The NRI assess the putative clinical utility of different non-invasive measures (in addition to TRFs) as a marker of CV event occurrence or mortality. The IDI can be viewed as a continuous version of the NRI and does not require predefined risk categories; only the predicted probability of an event is used in the reclassification. According to a receiver operating characteristic curve built for the composite endpoint, the best cutoff for the coronary artery calcification score was 259.5 AU (with a sensibility of 0.71 and a specificity of 0.73) and the best cutoff for the aortic calcification score was 1.32% (with a sensibility of 0.735 and a specificity of 0.61).

The threshold for statistical significance was set to p≤0.05. All statistical analyses were performed using SPSS software (version 18.0, SPSS Inc., Chicago, IL) for Windows (Microsoft Corp, Redmond, WA) and SAS software (version 9.2, SAS Institute Inc., Cary, NC).

## Results

**Fig 1:**
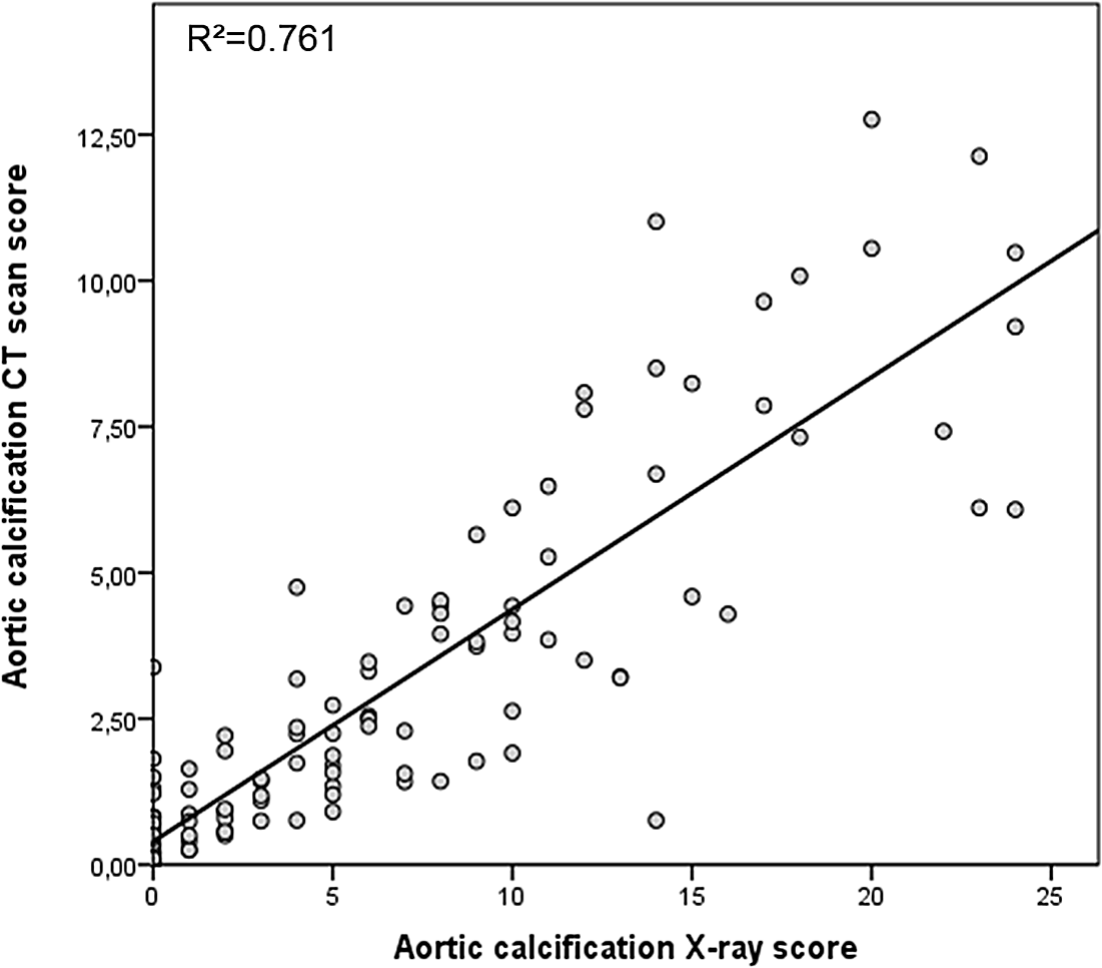
Linear regression curve. Relationship between the aortic calcification scores based on a CT scan or on X-ray data (n = 143, r^2^ = 0.761, p>0.001).

The main characteristics of the 143 CKD patients (grouped according to the occurrence or non-occurrence of the composite outcome, i.e. a CV event or death) are summarized in Table 1. The recorded causes of kidney disease were as follows: diabetic nephropathy (in 21% of the patients), nephroangiosclerosis and glomerulonephritis (18%), interstitial nephropathy (7%), hypertensive nephropathy (5%), polykystosis (5%) and other recorded diagnoses (hereditary disease, solitary kidney, dysglobulinemia, and systemic disease) (22%). There was no known cause in 22% of cases. When compared with event-free patients, patients with events were significantly older and were more likely to be at later CKD stages, be on dialysis or have a history of CV disease. Patients with events also had a lower diastolic blood pressure and lower albumin, hemoglobin, 1.25 (OH)_2_ vitamin D and 25 (OH) vitamin D levels (relative to event-free patients). Moreover, patients with events had higher aortic and coronary artery calcification scores (according to CT and X-ray data) and IMT and PWV values than event-free patients. There was no significance difference between the two groups in terms of the ABI or concomitant treatments. We observed a significant, linear relationship between the aortic calcification score (as evaluated in the CT scan) and the aortic calcification score (as evaluated from X-rays) (p<0.0001) (Fig 1). All non-invasive measurements were intercorrelated (except for the ABI, which was not correlated with any of the other measurements) (Table 2).

**Table 1:** Main demographic, biochemical and clinical characteristics, as a function of outcome.

	No event (n = 79)	Events(n = 64)	p
**Age, years**	**64 ± 12**	**70 ± 12**	**0.001**
Male gender, n (%)	43 (54.4)	44 (68.8)	0.088
BMI, kg/m^**2**^	28.6 ± 6	28 ± 7	0.565
Diabetes status, n (%)	32 (40.5)	29 (45.3)	0.612
Smoking status, n (%)	9 (11.5)	8 (12.9)	0.801
Systolic blood pressure, mmHg	151 ± 24	156 ± 29	0.256
**Diastolic blood pressure, mmHg**	**83 ± 11**	**79 ± 14**	**0.036**
ABI	1.14 ± 0.3 (1.09; 1–1.18)	1.22 ± 0.5 (1.09; 0.96–1.39)	0.266
**History of CV disease, n (%)**	**18 (22.8)**	**27 (42.2)**	**0.018**
**Pulse wave velocity, m/s**	**13.7 ± 3.4**	**15.7 ± 4.12**	**0.003**
**Dialysis, n (%)**	**19 (24.1)**	**28 (43.8)**	**0.019**
**CKD stage, n (%)**			**0.024**
2	11 (13.9)	1 (1.6)	
3	22 (27.8)	15 (23.4)	
4	22 (27.8)	15 (23.4)	
5	5 (6.3)	5 (7.8)	
5D	19 (24.1)	28 (43.8)	
**Aortic calcification CT scan score, %**	**2.1 ± 2.3(1.2; 0.5–3.4)**	**4.2 ± 3.5 (3.2; 1.6–7.2)**	**< 0.001**
**Coronary artery calcification score, AUs**	**358 ± 629(94; 15–354)**	**1018 ± 1784 (602; 131–983)**	**0.011**
**Aortic calcification X-ray score**	**4.6 ± 5.8 (2; 0–8)**	**8.4 ± 6.9 (6; 3–13)**	**0.001**
**Intima media thickness, mm**	**0.91 ± 0.15**	**0.98 ± 0.17**	**0.010**
*Concomitant medication*			
ACEI	13 (20.3)	15 (19)	0.844
ARB	37 (57.8)	49 (62)	0.137
Beta-blockers	32 (40.5)	31 (48.4)	0.347
Diuretics	26 (40.6)	42 (53.2)	0.613
Calcium antagonist	31 (39.2)	26 (40.6)	0.868
Vitamin D supplementation	29 (36.7)	22 (34.4)	0.774
Sevelamer	10 (12.7)	16 (25.0)	0.065
ESA	24 (30.4)	29 (45.3)	0.069
Lipid lowering therapy	34 (53.1)	53 (67.1)	0.092
Calcium, mmol/l	2.3 ± 0.2	2.3 ± 0.2	0.167
Phosphate, mmol/l	1.23 ± 0.42 (1.15; 1.03–1.38)	1.35 ± 0.49(1.26; 1.02–1.63)	0.127
LDL cholesterol, mmol/l	2.6 ± 0.9	2.7 ± 0.9	0.762
Total cholesterol, mmol/l	4.9 ± 1.2	4.9 ± 1.2	0.815
Triglycerides, mmol/l	2.1 ± 1.6 (1.6; 1.2–2.6)	2.1 ± 1.1 (1.8; 1.2–2.6)	0.857
**Albumin, g/l**	**38.9 ± 6.4**	**35.5 ± 5.7**	**0.001**
**eGFR (predialysis patients n = 96)**	**38 ± 19 (n = 60)**	**30 ± 17 (n = 36)**	**0.035**
Intact parathyroid hormone, pg/ml	145 ± 163 (80; 42–193)	126 ± 96 (87; 52–173)	0.394
**25 (OH) vitamin D, ng/ml**	**22.4 ± 15.4 (17.7; 11.9–31.4)**	**17.9 ± 10.3 (16.1; 9.8–21.3)**	**0.042**
**1,25 (OH)**_**2**_ **vitamin D, pg/ml**	**13.4 ± 10.1 (12; 4.3–17)**	**8.9 ± 10.2 (5; 2.5–17.5)**	**0.035**
C-reactive protein, mg/l	8.8 ± 22.5 (2.3; 1.1–6.6)	14.1 ± 25.4 (4.0; 1.5–13.9)	0.186
Interleukin 6, pg/ml	4.0 ± 5.1 (2.1; 0.9–5.8)	6.6 ± 9.9 (3.8; 1.9–7.0)	0.710
**Hemoglobin, g/dl**	**12.4 ± 1.8**	**11.7 ± 1.7**	**0.018**

Abbreviation: BMI: body mass index; ABI: ankle-brachial index; CKD: chronic kidney disease, AUs: Agatston units, ACEI: angiotensin-converting-enzyme inhibitors, ARB: Angiotensin receptor blockers, ESA: erythropoietin stimulating agent, LDL: low-density lipoprotein.

CKD stages were evaluated according to the glomerular filtration rate estimated with the CKD-EPI equation.

Non-Gaussian variables are expressed as the mean ± SD (median; 25^th^-75^th^ quartiles)

**Table 2:** Correlation between non-invasive measurements.

		IMT	PWV	Aortic calcification score (CT scan)	Aortic calcification score (X-ray)	Coronary artery calcification	ABI
IMT	r		**0.334**	**0.318**	**0.037**	**0.258**	-0.159
	p value		**<0.001**	**0.001**	**<0.001**	**0.019**	0.100
PWV	r	**0.334**		**0.453**	**0.411**	**0.449**	-0.071
	p value	**<0.001**		**<0.001**	**<0.001**	**<0.001**	0.428
Aortic calcification score (CT scan)	r	**0.318**	**0.453**		**0.875**	**0.644**	-0.051
	p value	**0.001**	**<0.001**		**<0.001**	**<0.001**	0.583
Aortic calcification score (X-ray)	r	**0.037**	**0.411**	**0.875**		**0.544**	-0.078
	p value	**<0.001**	**<0.001**	**<0.001**		**<0.001**	0.410
Coronary artery calcification	r	**0.258**	**0.449**	**0.644**	**0.544**		0.010
	p value	**0.019**	**<0.001**	**<0.001**	**<0.001**		0.929
ABI	r	-0.159	-0.071	-0.051	-0.078	0.010	
	p value	0.100	0.428	0.583	0.410	0.929	

Abbreviations: IMT: intima-media thickness; PWV: pulse wave velocity; ABI: ankle brachial index.

**Fig 2:**
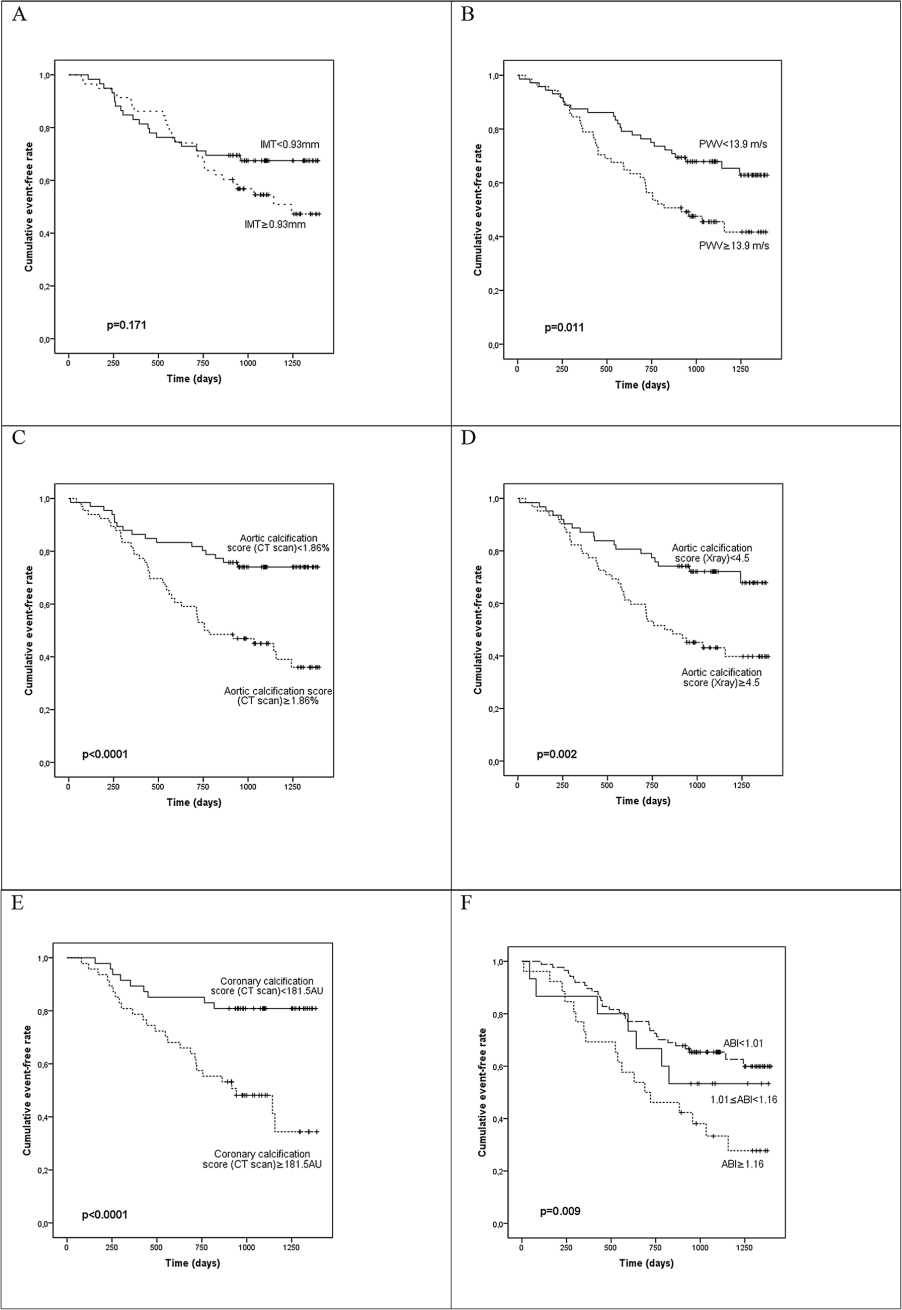
Unadjusted Kaplan-Meier cumulative event curves for all-cause mortality and the first non-fatal CV event, as a function of the median value of the (A) IMT, (B) PWV, (C) aortic calcification score (CT scan), (D) aortic calcification score (X-ray), (E) coronary artery calcification score (CT scan), and ABI tertile (F).

During the follow-up period (mean duration: 873 ± 393 days), 44 patients died (24 from cardiovascular causes, from 12 infectious diseases and 8 from other causes) and 30 patients had CV events leading to 64 composite endpoints for analysis. In a crude analysis (Fig 2), a high PWV, a high aortic calcification score (according to the CT scan or X-rays) and a high coronary artery calcification score were all significant predictors of overall mortality and CV events (p = 0.011, p<0.001, p = 0.002 and p<0.001, respectively, in a log-rank test comparing the survival curves). Dividing the population into ABI tertiles revealed that patients with a higher ABI had a greater relative risk for outcomes (p = 0.009) (Fig 2F).

The various Cox regression models (Table 3) confirmed that after adjustment for TRFs, only a high aortic calcification score (evaluated from the CT scan) and a high coronary artery calcification score were good predictors of all-cause mortality and CV events.

**Fig 3:**
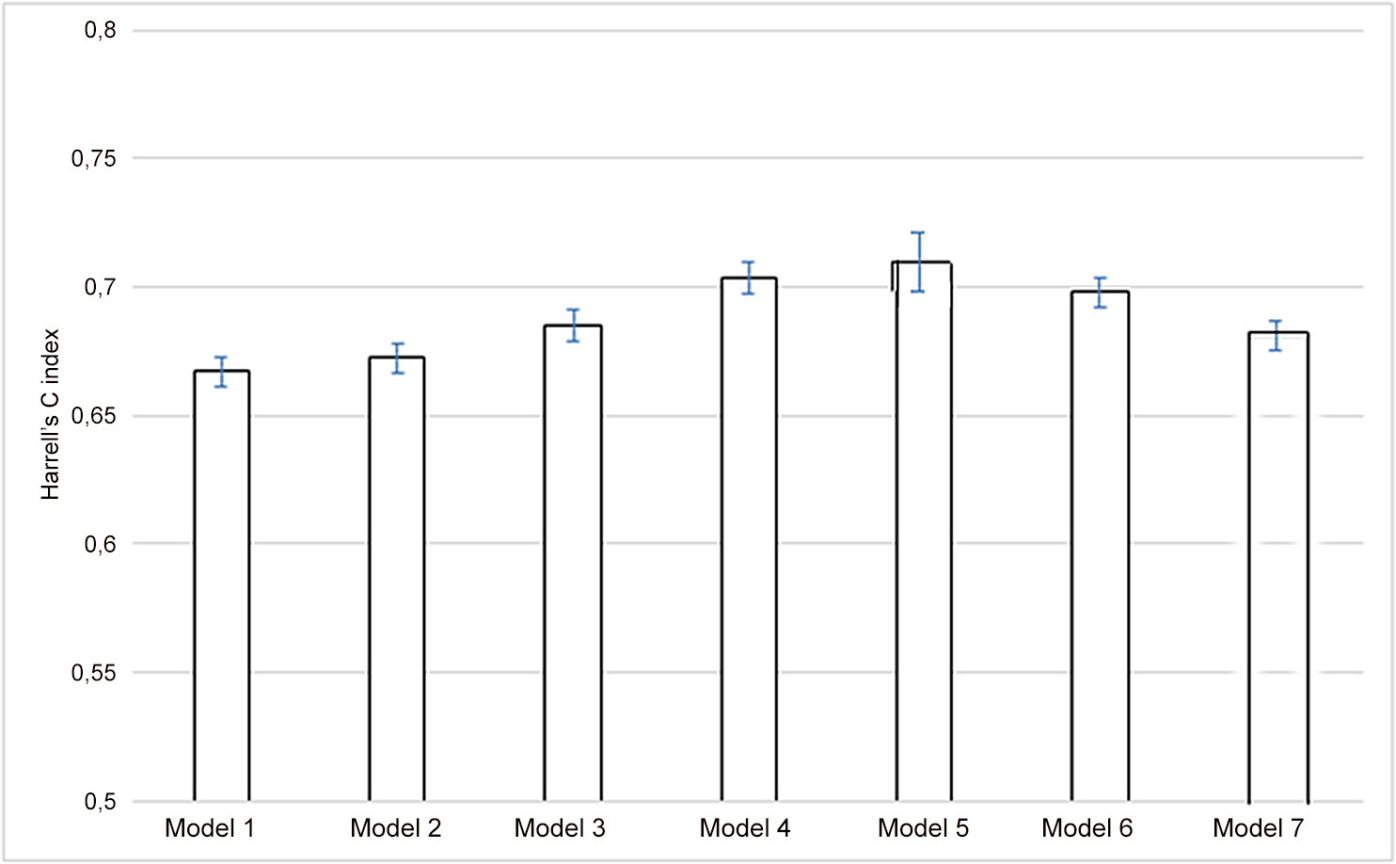
Harrell’s C-index for the various Cox models. Model 1, traditional risk factors (TRFs): 0.67 ± 0.35; Model 2, TRFs + PWV: 0.67 ± 0.036; Model 3, TRFs + IMT: 0.69 ± 0.036; Model 4, TRFs + aortic calcification score (CT scan): 0.70 ± 0.036; Model 5, TRFs + aortic calcification score (X-ray): 0.71 ± 0.05; Model 6, TRFs + coronary artery calcification score: 0.70 ± 0.036; Model 7, TRFs + ABI (<1.3 vs. >1.3) 0.69 ± 0.035. All differences were non-significant.

The adjusted Cox models' respective abilities to predict outcome with the different non-invasive methods are presented in Fig 3. Even though there was a trend towards an improvement in Harrell’s C-index (especially with calcification score: models 4, 5 and 6), the difference was not statistically significant. Furthermore, we failed to observe a significant improvement in Harrell’s C-index when combining the various non-invasive measurements with TRFs.

**Table 3:** Multivariate Cox regression analysis of risk factors at baseline for all-cause mortality and first non-fatal CV events.

*Models of patient survival (events*: *n = 64)*	RR	95%CI	p
*Model 1*: *TRFs*			
**Age**	**1.047**	**1.021–1.073**	**<0.001**
**Gender (female vs. male)**	**0.554**	**0.319–0.962**	**0.036**
Triglycerides	0.975	0.822–1.157	0.776
Diabetes	0.969	0.514–1.828	0.923
Hypertension	0.600	0.267–1.350	0.217
Smoking status	1.505	0.657–3.451	0.334
*Model 2*: *TRFs + PWV*			
**Age**	**1.038**	**1.010–1.067**	**0.007**
**Gender (female vs. male)**	**0.577**	**0.331–1.000**	**0.050**
Triglycerides	0.973	0.813–1.165	0.767
Diabetes	0.913	0.483–1.726	0.779
Hypertension	0.601	0.267–1.350	0.217
Smoking status	1.535	0.671–3.510	0.310
PWV	1.070	0.988–1.159	0.097
*Model 3*: *TRFs + IMT*			
**Age**	**1.053**	**1.018–1.088**	**0.002**
**Gender (female vs. male)**	**0.483**	**0.243–0.960**	**0.038**
Triglycerides	0.980	0.817–1.174	0.823
Diabetes	0.874	0.409–1.869	0.728
Hypertension	0.989	0.298–3.280	0.985
Smoking status	1.081	0.361–3.241	0.889
IMT	0.699	0.085–5.775	0.739
*Model 4*: *TRFs + aortic calcification score (CT scan)*			
**Aortic calcification score**	**1.161**	**1.048–1.287**	**0.004**
**Gender (female vs. male)**	**0.542**	**0.295–0.996**	**0.048**
**Hypertension**	**2.425**	**1.034–5.690**	**0.042**
Age	1.020	0.990–1.051	0.198
Diabetes	1.003	0.491–2.050	0.992
Triglycerides	0.959	0.794–1.158	0.660
Smoking status	1.009	0.396–2.574	0.985
Model 5: *TRFs + aortic calcification score (X-ray)*			
**Age**	**1.055**	**1.021–1.089**	**0.001**
**Gender**	**0.462**	**0.251–0.849**	**0.013**
Diabetes	0.887	0.442–1.782	0.737
Hypertension	0.395	0.157–0.995	0.062
Triglycerides	0.937	0.773–1.136	0.509
Smoking status	1.134	0.364–3.527	0.828
Aortic calcification score (X-ray)	1.029	0.989–1.071	0.153
*Model 6*: *TRFs + coronary artery calcification score (in 100 AU increments)*			
**Coronary artery calcification score**	**1.024**	**1.005–1.043**	**0.012**
**Age**	**1.036**	**1.002–1.071**	**0.036**
Gender	0.830	0.370–1.860	0.650
Diabetes	0.772	0.285–2.088	0.610
Hypertension	0.382	0.127–1.152	0.088
Triglycerides	1.000	0.811–1.233	0.998
Smoking Status	2.591	0.906–7.404	0.076
*Model 7*: *TRFs + ankle brachial index (<1*.*3 vs*. *>1*.*3)*			
**Age**	**1.050**	**1.023–1.079**	**<0.001**
**Diabetes**	**2.285**	**1.213–4.303**	**0.011**
Gender	0.640	0.357–1.145	0.133
Hypertension	0.610	0.253–1.473	0.272
Triglycerides	0.980	0.815–1.177	0.826
Smoking status	1.546	0.641–3.732	0.332
Ankle brachial index	1.483	0.756–2.908	0.252

IMT: intima media thickness; PWV: pulse wave velocity; RR: risk ratio; CI: confidence interval. TRFs (traditional risk factors): age (in 1-year increments), gender, diabetes, triglyceride level (in 1 mmol/l increments), hypertension, and smoking status. Coronary calcification severity was considered in 100 AU increments.

The NRI was calculated in order to assess the putative clinical utility of the aortic calcification score (evaluated from the CT scan) and the coronary artery calcification score as markers of CV risk when combined with TRFs (Table 4). A significantly positive IDI (which represents a continuous measure and does not rely on prespecified risk categories) was observed for the coronary artery calcification score but not for the aortic calcification score. A significantly positive categorical NRI was observed only for the coronary artery calcification score; this was mainly due to greater specificity (NRI for non-events: 0.2 (0.06; 0.34).

**Table 4:** Improvement in prediction of the composite endpoint by adding the aortic calcification score and the coronary artery calcification score to TRFs.

Predictors	IDI (95% CI)	NRI (95% CI)	
		Event	Nonevent
aortic calcification score (CT scan)	0.04 (-0.004;0.075)	0.05 (-0.02;0.12)	0.06 (-0.04;0.16)
coronary artery calcification score	**0.08 (0.001–0.149)**	0.08 (-0.03;0.19)	**0.2 (0.06;0.34)**

Abbreviations: CI: confidence interval; NRI: net reclassification improvement; IDI: integrated discrimination improvement.

## Discussion

At present, only TRFs are routinely evaluated as predictors of CV events, CV mortality and all-cause mortality. Some studies have shown that addition of a non-invasive measurement to these TRFs improves the predictive value in the general population and in CKD patients [20, 21, 32, 33]. The objective of the present study was to determine whether the addition of non-invasive methods (such as the IMT, PWV, ABI and vascular calcification score) to TRFs (such as age, gender, hypertension, diabetes, smoking status and high triglyceride levels) improves prediction of the CV risk in patients at different CKD stages. In a cohort of stage 2–5D CKD patients, our results showed that PWV, aortic/coronary artery calcification scores and the ABI predict all-cause mortality and CV events in a univariate analysis. However, only the aortic and coronary artery calcification scores appear to be independent predictors in multivariate models. Hence, evaluation of vascular calcification scores (and especially the coronary artery calcification score) provides additional predictive power for the CV risk, when compared with TRFs alone.

It is noteworthy that in our study population, the IMT and PWV did not have predictive value for CVD events and mortality–even after adjustment for TRFs (Table 2, models 2 and 3). In contrast to our present results, Karras et al. reported that PWV measurement was able to predict the outcome of patients with stage 2 to 5 CKD both in terms of overall survival and the occurrence of fatal or non-fatal CV events [13]. This disparity might be due to inter-study differences in PWVs, with mean values of 14.7 m/s (showing arterial stiffness) in our study population and 11.9 m/s in Karras’ study population. It appears that the patients in Karras et al.’s study were generally healthier (as shown by the lower mean PWV) that those in the present study, although the ranges of PWV were broader in the former study. In terms of the IMT evaluation, Zoungas et al.'s study of a cohort of 315 patients with stage 4 to 5 CKD found that the aortofemoral PWV (but not the carotid arterial IMT) was independently associated with the CV outcome [34]. Furthermore, the disparities between these studies may be due to differences in the statistical power (the sample size) and the CKD severity.

In contrast, a study of 438 hemodialysis patients with end-stage renal disease reported that carotid artery IMT is an independently predictor of CV mortality [10]. This non-invasive method seems to be a better predictor of CV mortality in dialysis patients than in pre-dialysis patients.

It is important to note that the evaluation of vascular calcification adds new information for the prediction and thus the potential prevention of all-cause mortality or CV events. Indeed, patients with an elevated aortic/coronary artery calcification score have an elevated risk of mortality and CV events—regardless of TRFs. Moreover, our evaluation of the coronary artery calcification score increased the specificity of risk reclassification of CKD patients. We also observed a very strong correlation between the two radiologic methods used to derive the aortic calcification scores (X-rays and a CT scan). The X-ray-based evaluation of aortic calcification is a relative simple tool and could be easily performed routinely in CKD populations. Matsushita et al. evaluated a number of methods (IMT, the ABI and a coronary artery calcium score) likely to improve CV risk prediction in 6553 multi-ethnic participants (including subjects with CKD) [20]. In Cox proportional hazards models adjusted for Framingham predictors, each subclinical measure was independently associated with CV outcomes. Moreover, inclusion of the coronary artery calcium score led to greater increases in (i) the C statistic for predicting CV disease and (ii) the NRI. As in our present study, Matsushita et al. found that the coronary artery calcium score produced the greatest improvement in cardiovascular risk prediction. However, Matsushita et al. also mentioned that the evaluation of coronary artery calcification raises several issues, including cost effectiveness, exposure to ionizing radiation and access to CT facilities in low-income countries. Hence, an X-ray-based aortic calcification score may be a good alternative to CT. However, the value of the aortic calcification score need to be confirmed; in the present work, the score appeared to be a good predictor when using Cox multivariate models but not using Harrell's C-index or NRI—probably due to a lack of power or to differences that could be detected in the Cox model but were too small for the Harrell's C-index or the NRI analysis.

Our study had some limitations, including the relatively small cohort size, the relatively small number of CV events and deaths and the lack of data on proteinuria. However, we used composite criteria to overcome this limitation. In contrast, the study's main strength relates to the simultaneous use of several methods to evaluate atherosclerosis/arteriosclerosis.

In conclusion, we have highlighted a simple, non-invasive diagnostic tool that may be capable of improving CV risk assessment (when compared with TRFs alone) in patients with CKD. Patients with high vascular calcification scores (and especially a high coronary artery calcification score) can be considered as having the highest CV risk. However, the present results should be confirmed in larger cohorts.
